# Integrative Analysis and Machine Learning based Characterization of Single Circulating Tumor Cells

**DOI:** 10.3390/jcm9041206

**Published:** 2020-04-22

**Authors:** Arvind Iyer, Krishan Gupta, Shreya Sharma, Kishore Hari, Yi Fang Lee, Neevan Ramalingam, Yoon Sim Yap, Jay West, Ali Asgar Bhagat, Balaram Vishnu Subramani, Burhanuddin Sabuwala, Tuan Zea Tan, Jean Paul Thiery, Mohit Kumar Jolly, Naveen Ramalingam, Debarka Sengupta

**Affiliations:** 1 Department of Computational Biology, Indraprastha Institute of Information Technology, New Delhi 110020, India; arvind16122@iiitd.ac.in; 2 Department of Computer Science and Engineering, Indraprastha Institute of Information Technology, New Delhi 110020, India; krishang@iiitd.ac.in (K.G.); shreya15096@iiitd.ac.in (S.S.); 3 Centre for BioSystems Science and Engineering, Indian Institute of Science, Bangalore 560012, India; kishorehari@iisc.ac.in (K.H.); mkjolly@iisc.ac.in (M.K.J.); 4 Biolidics Limited, 81 Science Park Drive, 02-03 The Chadwick, Singapore 118257, Singapore; yifang@biolidics.com (Y.F.L.); ali@nus.edu.sg (A.A.B.); 5 Qualcomm Incorporated, 5775 Morehouse Drive, San Diego, CA 92121, USA; neevan.snap@gmail.com; 6 National Cancer Centre Singapore, 11 Hospital Dr, Singapore 169610, Singapore; Yap.Y.S@nccs.com.sg; 7 Fluidigm Corporation, 2 Tower Place, Suite 2000, South San Francisco, CA 94080, USA; drjayman@gmail.com; 8 School of Mathematics, Indian Institute of Science Education and Research, Thiruvananthapuram 695551, India; balaram.vishnu17@iisertvm.ac.in; 9 Department of Biotechnology, Indian Institute of Technology Madras, Chennai 600036, India; be17b011@smail.iitm.ac.in; 10 Cancer Science Institute of Singapore, National University of Singapore, Center for Translational Medicine, Singapore 117599, Singapore; csittz@nus.edu.sg; 11 Guangzhou Regenerative Medicine and Health; Guangdong laboratory, Chinese Academy of Science, Guangzhou 510530, China; bchtjp@nus.edu.sg; 12 Center for Artificial Intelligence, Indraprastha Institute of Information Technology, New Delhi 110020, India

**Keywords:** high-throughput sequencing, rare cell type, single-cell, RNA-seq, machine learning, CTC, blood

## Abstract

We collated publicly available single-cell expression profiles of circulating tumor cells (CTCs) and showed that CTCs across cancers lie on a near-perfect continuum of epithelial to mesenchymal (EMT) transition. Integrative analysis of CTC transcriptomes also highlighted the inverse gene expression pattern between PD-L1 and MHC, which is implicated in cancer immunotherapy. We used the CTCs expression profiles in tandem with publicly available peripheral blood mononuclear cell (PBMC) transcriptomes to train a classifier that accurately recognizes CTCs of diverse phenotype. Further, we used this classifier to validate circulating breast tumor cells captured using a newly developed microfluidic system for label-free enrichment of CTCs.

## 1. Introduction

A staggering 90% of cancer deaths are attributable to metastases [1]. After detaching from solid tumors, cancer cells travel through the bloodstream to reach distant organs and seed the development of metastatic tumors [2]. Cancer cells under circulation are called circulating tumor cells (CTCs) [3]. As a blood-based bio marker, CTCs offer unabated, real-time insights into tumor evolution and therapeutic responses. Despite these promises, the rareness of CTCs in the peripheral blood hinders their isolation and characterization [3]. Cancers in solid tissues develop from epithelial cells, which are typically densely packed in layers. However, dissemination and migration of cancer cells during metastasis require the acquisition of mesenchymal-like features. Transcendence of epithelial cancer cells into mesenchymal-like ones is popularly known as Epithelial to Mesenchymal Transition (EMT).

It is widely understood that due to the loss of epithelial property only a fraction of CTCs can be expected to express canonical epithelial markers such as Epithelial Cell Adhesion Molecule (*EpCAM*). The only FDA (Food and Drug Administration) approved CTC capture platform CELLSEARCH^®^ uses epithelial surface marker *EpCAM* to detect CTCs in patients blood [4]. Controlled experiments involving cell-lines have shown that recovery of cells with *EpCAM* expression varies a lot and many canonical epithelial markers are down-regulated in CTCs, undergoing epithelial-mesenchymal transition (EMT) [5]. Therefore, marker-based enrichment techniques are sub-optimal for the comprehensive charting of heterogeneous CTC sub-populations. [6,7,8] Over the past few years, various CTC capture platforms exploiting biophysical characteristics of cancer cells have been developed [9,10,11]. *CD45*-based negative enrichment has also been adopted as an alternative strategy. The potential of such antigen-agnostic platforms have not been fully utilized since the chances of immune cell contamination cannot be completely ruled out [9,10]. The recent advent of single-cell RNA sequencing (scRNA-seq) has allowed molecular profiling of single CTCs [12], captured using microfluidic devices [13,14,15,16,17]. Almost all studies that reported molecular profiles of single CTCs resorted to marker based bioinformatic annotation of cell types or applied post-capture staining of CTCs using epithelial/cancer-specific molecular markers [13,18]. To broad base the detection of CTCs, it is therefore important that we develop a scheme to recognize diverse CTC phenotypes presented within a large pool of immune cells.

In this study, we report the ClearCell^®^ Polaris^™^ workflow that employs size-dependant enrichment of CTCs, followed by negative selection for *CD45* [14,19]. For unbiased labeling of cells of cancer origin, we use publicly available single-cell expression profiles of CTCs and Peripheral Blood Mononuclear Cells (PBMCs) to train a classification system that reliably recognizes a wide variety of CTCs from across different cancer types. In summary, we propose a strategy to employ machine learning based models to detect CTCs retrieved using marker agnostic microfluidic technologies.

## 2. Materials and Methods

### 2.1. Description of Datasets

We collected single-cell RNA-seq (scRNA seq) data of circulating tumor cells (CTCs) and peripheral blood mononuclear cells (PBMCs) from 14 different studies in total [2,13,18,20,21,22,23,24,25,26,27,28] We acquired 558 single CTCs from 10 of these 14 studies. On the other hand, 6 of these studies supplied a total of 37665 PBMCs. Two of these studies with accession numbers GSE67980 and GSE109761 respective offer both blood and CTC transcriptomes. The CTC data entailed five cancer types breast, prostate, melanoma, lung, and pancreas. Notably, circulating breast tumor cells in the data was supplied by six different studies. Remaining cancer types were represented by single studies (Supplementary Table S1).

### 2.2. Data Pre-Processing

We downloaded raw read count data for every study from their respective sources (Supplementary Table S1). While merging, we found 15,043 genes common across all the datasets. First, we discarded the poor quality cells that had less than 10% of the genes having non zero expression. The filtering step retained about 5% (1861) of the input cells. Genes with count ≥5 in at least 10 cells were retained. A total of 12,335 genes were left after this. Among the 1861 cells, 538 were CTCs. Our final data contained a 12,335 expressed genes and 1861 cells, of which 538 were CTCs. At this stage, we standardized the library depths using median normalization [29,30,31]. The expression matrix thus obtained was log-transformed after the addition of 1 as pseudo-count. Different gene selection techniques and data used for the various downstream analyses are mentioned in the subsequent sections.

### 2.3. Construction of Epithelial and Mesenchymal Signatures and E:M Score

While integrating CTC datasets alone, we found 17609 genes common across all 558 CTCs coming from 10 publicly available CTC datasets (Supplementary Table S1). We retained CTCs that expressed at least 5% of the 17609 genes. Genes with read count >5 in at least 10 CTCs were considered for further analyses. At this stage we were left with an expression matrix consisting of 13,600 genes and 554 CTCs. We constructed a panel of 176 well-known epithelial, mesenchymal, and cancer stem cell markers combining information from the CellMarker database [29] and existing literature. The expression matrix of marker genes thus obtained was subjected to stricter criteria for gene and cell selection. We retained 550 cells that expressed at least 10% of these marker genes. Marker genes having minimum read count >5 in at least 30% of these cells were selected for the subsequent analyses. The resulted matrix consisted of 550 cells and 81 marker genes (16 epithelial, 39 mesenchymal, and 26 cancer stem cell markers, see (Supplementary Table S2). We median normalized and log-transformed the generated matrix. For each cell, we computed a comprehensive score for both epithelial and mesenchymal phenotype. To compute the score we first applied Z-score transformation on each cell. To create the signature for specific phenotype, for each cell we combined Z-transformed marker expressions using the below formula.

(1) $$Z_{phenotype}=\frac{\sum_{\forall i\in markers}Z_{i}}{\sqrt[{}]{\left|markers\right|}}$$

Here $Z_{phenotype}$ is a comprehensive phenotype specific score computed over individual Z-transformed marker expressions denoted by $Z_{i}$, where markers denotes the set of markers corresponding to the concerned phenotype. We assigned each single CTC an E:M score by computing the ratio between $Z_{phenotypes}$ computed for epithelial and mesenchymal genes respectively.

### 2.4. Simulation of E-M Continuum

We identified the regulatory interactions among epithelial (E) and mesenchymal (M) genes under study, together with their connections to canonical regulators of EMT and MET such as the double negative feedback loops involving *miR-200, ZEB and GRHL2* (Supplementary Note-1). For the constructed network, an ensemble of mathematical models were then created using RACIPE (RAndom CIrcuit PErturbation), which considers a set of kinetic parameters randomly chosen from within the biologically relevant ranges [30]. This helps to identify the robust gene expression signatures that can emerge due to given network topology. The simulations were performed in triplets to avoid numerical artifacts/variations due to random sampling. Such an ensemble of models is usually based on ordinary differential equations (ODEs), such as the one mentioned below.

$$\frac{d[VIM]}{dt}=l_{VIM}H^{S+}\left(ZEB,VIM\right)H^{S-}\left(STEP1,VIM\right)-k_{VIM}\left[VIM\right]$$

where [VIM] is the concentration of VIM, and $l_{VIM}$ and $k_{VIM}$ are its production and degradation rates respectively. $H^{S+}\left(X,Y\right)$/ $H^{S-}\left(X,Y\right)$ are the shifted Hill functions that result in up-regulation/down-regulation caused in the expression of Y due to X.

### 2.5. Classification of Cancer and Blood Transcriptomes

To model the phenotypic identities of CTCs and PBMCs, we trained various classification models. To broad-base our feature selection we used about 3000 cell-type specific markers (Supplementary Table S3) reported in the CellMarker database [29]. Besides, the median normalization we subjected the data to principal component analysis (PCA) [31] and also applied harmony batch correction method [32]. We used three popular classification techniques - Naive Bayes (NB) [33], Gradient Boosting Machines (GBM) [34] and Random Forest (RF) [35] on the training datasets. We evaluated the model on five different datasets: 1. Clearcell-Polaris CTCs; 2. Hydro-Seq Data which uses a novel, hydrodynamic scRNA-seq barcoding technique, for high-throughput CTC capture [11]; 3. the leftover PBMCs, not used for model training; 4. a combination of Clearcell-Polaris and randomly sampled unused 500 PBMC expression profiles; and 5. a combination of Hyrdo-seq data and randomly sampled unused 500 PBMC expression profiles. We computed the accuracy percentage using the equation:

(2) $$Accuracy=\frac{\left(TP+TN\right)}{\left(TP+TN+FP+FN\right)}$$

Besides the accuracy percentage, we reported additional model evaluation metrics such as F1 score, Mathews correlation coefficient (MCC) and Cohen’s kappa as applicable (Supplementary Table S4).

### 2.6. Sample Collection

Blood specimens of three *HER2-* (Human epidermal growth factor receptor 2) breast cancer patients (identified as P3, P4, P5) were obtained from the National Cancer Center Singapore, with informed consent following the approved procedures under the institutional review board (IRB) guidelines (CIRB no. 2014/119/B). The clinical sample collection protocols were reviewed and approved by the Sing Health Centralised Institutional Review Board. The determination of estrogen receptor (*ER*), progesterone receptor (*PR*) and human epidermal growth factor receptor 2 (*HER2*) status by immunohistochemistry in this study was based on the latest recommendations of the American Society of Clinical Oncology and the College of American Pathologists. All three subjects had *ER+/PR+/HER2-* hormone receptor status as analyzed by immunohistochemistry. For P3, blood was drawn (baseline) in August 2016 for CTC enrichment. Following this P3 was on chemotherapy. P4 and P5 were on chemotherapy before their blood samples were collected for CTC enrichment in August and September of 2016, respectively.

### 2.7. CTC Enrichment

Blood samples were collected in 9 mL of K3EDTA blood collection tubes (Greiner Bio-One, 455036). 6–8.5 mL of whole blood was processed for each run. Red blood cells were first removed with the addition of red blood cell (RBC) lysis buffer (G-Bioscience, St. Louis, MO, USA) and incubation for 10 min at room temperature. Lysed RBCs in the supernatant were discarded after centrifugation. The nucleated cell pellet was suspended in a ClearCell resuspension buffer before CTC enrichment on the ClearCell FX system (Biolidics Limited) [36], performed following manufacturer’s instructions.

### 2.8. Immunofluorescence Suspension Staining

The enriched CTC blood sample was centrifuged at 300 g for 10 min and concentrated to 70 μL. The cells were stained with the addition of the following markers and antibodies for 1 hour: CellTracker Orange (CTO) (Thermo Fisher, C34551), Calcein AM (Thermo Fisher, L3224), CD45 antibody- conjugated with Alexa 647 (Bio Legend, 304020), and CD31- conjugated with Alexa 647 (Bio Legend, 303111). 15 μL of RPMI with 10% FBS (Gibco) and 3 μL of RNase inhibitor (Thermo Fisher, N8080119) were also added to improve the viability and RNA quality of the cells. After incubation, 13 mL of PBS was added to dilute the staining reagents. The sample was spun down at 300 g for 10 min and concentrated to 45 μL. In order to achieve optimal buoyancy in an integrated fluidic circuit (IFC), 45 μL of CTCs was mixed with a 30 μL Cell suspension Reagent (Fluidigm, 101-0434) to achieve 75 μL of cell mix.

### 2.9. Integrated Fluidic Circuit (IFC) Operation

The Polaris IFC is first primed using the Fluidigm Polaris system^TM^ [19] to fill the control lines on the fluidic circuit, load cell capture beads, and block the inside of PDMS channels to prevent non-specific absorption/adsorption of proteins. To capture and maintain the single cells in the sites, the capture sites (48 sites) are preloaded with beads that are linked on IFC to fabricate a tightly packed bead column during the IFC prime step. After completion of the prime step, the cell mix (cells with suspension reagent) is loaded in three inlets (25 μL each of cell mix) on the Polaris IFC and single cells with CTO+& Calcein AM+& CD45−& CD31− are selected to capture sites. Finally, the single cells are processed through template-switching mRNA-seq chemistry for full-length cDNA generation and preamplification on IFC.

### 2.10. mRNA-Seq Library Preparation and Sequencing

SMARTer^®^ Ultra^®^ Low RNA Kit for Illumina^®^ Sequencing (Clontech^®^, 634936) was used to generate preamplified cDNA. The selected and sequestered single cells were lysed using a Polaris cell lysis mixture. The 28-μL cell lysis mix consists of 8.0 μL of Polaris Lysis Reagent (Fluidigm, 101-1637), 9.6 μL of Polaris Lysis Plus Reagent (Fluidigm, 101-1635), 9.0 μL of 3 SMART™ CDS Primer II A (12 M, Clontech, 634936), and 1.4 μL of Loading Reagent (20X, Fluidigm, 101-1004). The thermal profile for single-cell lysis is 37 °C for 5 min, 72 °C for 3 min, 25 °C for 1 min, and hold at 4 °C. The 48-μL preparation volume for reverse transcription (RT) contains 1X SMARTer Kit 5X First-Strand Buffer (5X; Clontech, 634936), 2.5-mM SMARTer Kit Dithiothreitol (100 mM; Clontech, 634936), 1-mM SMARTer Kit dNTP Mix (10 mM each; Clontech, 634936), 1.2-μM SMARTer Kit SMARTer II A Oligonucleotide (12 μM; Clontech, 634936), 1-U/μL SMARTer Kit RNase Inhibitor (40 U/μL; Clontech, 634936), 10-U/μL SMARTScribe™ Reverse Transcriptase (100 U/μL; Clontech, 634936), and 3.2 μL of Polaris RT Plus Reagent (Fluidigm, 101-1366). All the concentrations correspond to those found in the RT chambers inside the Polaris IFC. The thermal protocol for RT is 42 °C for 90 min (RT), 70 °C for 10 min (enzyme inactivation), and a final hold at 4 °C.

The 90-μL preparation volume for PCR contains 1X Advantage 2 PCR Buffer [not short amplicon (SA)](10X, Clontech, 639206, Advantage^®^ 2 PCR Kit), 0.4-mM dNTP Mix (50X/10 mM, Clontech, 639206), 0.48-μM IS PCR Primer (12 μM, Clontech, 639206), 2X Advantage 2 Polymerase Mix (50X, Clontech, 639206), and 1X Loading Reagent (20X, Fluidigm, 101-1004). All the concentrations correspond to those found in the PCR chambers inside the Polaris IFC. The thermal protocol for preamplification consists of 95 °C for 1 min (enzyme activation), five cycles (95 °C for 20 s, 58 °C for 4 min, and 68 °C for 6 min), nine cycles (95 °C for 20 s, 64 °C for 30 s, and 68 °C for 6 min), seven cycles (95 °C for 30 s, 64 °C for 30 s, and 68 °C for 7 min), and final extension at 72 °C for 10 min. The preamplified cDNAs are harvested into 48 separate outlets on the Polaris IFC carrier. The cDNA reaction products were then converted into mRNA-seq libraries using the Nextera^®^ XT DNA Sample Preparation Kit (Illumina, FC-131-1096 and FC-131-2001, FC-131-2002, FC-131-2003, and FC-131-2004) following the manufacturer’s instructions with minor modifications. Specifically, reactions were run at one-quarter of the recommended volume, the tagmentation step was extended to 10 min, and the extension time during the PCR step was increased from 30 to 60 s. After the PCR step, samples were pooled, cleaned twice with 0.9× Agencourt AMPure XP SPRI beads (Beckman Coulter), eluted in Tris + EDTA buffer and quantified using a high-sensitivity DNA chip (Agilent). The pooled library was sequenced on Illumina MiSeq™ using reagent kit v3 (2 × 75 bp paired-end read). The sequencing data generated were processed by standard bioinformatics pipeline (Supplementary Note 2).

### 2.11. Reference Component Analysis of CTCs and PBMCs

For reference component analysis (RCA), we used the global panels supplied as part of the RCA R package [37]. Each of the global panels consisted of numerous tissue samples. RCA [37] uses cell type specific genes for measuring the correlation between the tissue types and the input single cells. Due to the low amount of starting RNA, single cell expression data is far noisier than bulk expression data. As a result, tissue types represented by lowly expressed feature genes can potentially give rise to significant levels of noise. In each global panel, we, therefore, retained 50% of the tissue types with the highest median expression of the feature genes. RCA [37] analysis provided us with both single cell-tissue correlation heat-map and 2D projection of the individual transcriptomes.

### 2.12. Data and Code Availability

The data-set used in the study are available from links mentioned in the (Supplementary Table S1). Single cell sequencing data generated for this paper is deposited at GEO with accession number GSE129474. Code used for analysis is available at this link and a R package is available at link.

## 3. Results

### 3.1. Integration of Single Cell Expression Datasets of Circulating Tumor Cells

**Figure 1:**
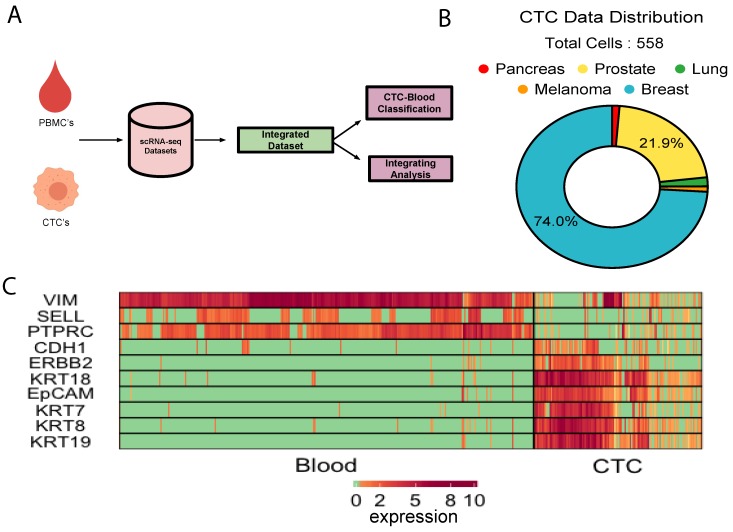
Integrative analysis of CTC transcriptomes: (**A**)Schematic of study. (**B**) Cancer types represented by the integrated CTC population. (**C**) Expression of canonical epithelial and immune cell markers in CTCs and the PBMCs under study.

We collected about 500 single CTC transcriptomes from 10 independent studies, representing five different cancer types i.e., breast, prostate, lung, pancreas, and melanoma (Figure 1B, Supplementary Table S1). On the other hands, as control, expression profiles of human PBMCs were collected from six different studies (Supplementary Table S1). About 70% of the CTCs came from various breast cancer studies. CTC datasets that we curated were of variable quality. We preprocessed the data to ensure that the poor-quality cells and unexpressed genes were discarded (Methods, Supplementary Figure S1). We further normalised the combined expression matrix to control for the library depth (Methods). We tracked expression of some of the canonical epithelial (*KRT8, KRT18, EpCAM, CDH1*) and leukocyte markers *(PTPRC, VIM)* to cross-validate the cell type identities. Elevated expression levels of a subset of epithelial markers were observed in a vast majority of the CTCs (Figure 1C, Supplementary Figure S2). Significant up-regulation of platelet and fibroblast markers was observed in large fractions of CTCs (Figure 1C, Supplementary Figure S2). This combined data source served as the basis for the majority of our analysis and development of the CTC-immune cell classification system (Figure 1A).

### 3.2. Ubiquity of Epithelial-Mesenchymal Transition in Cancer Metastasis

**Figure 2:**
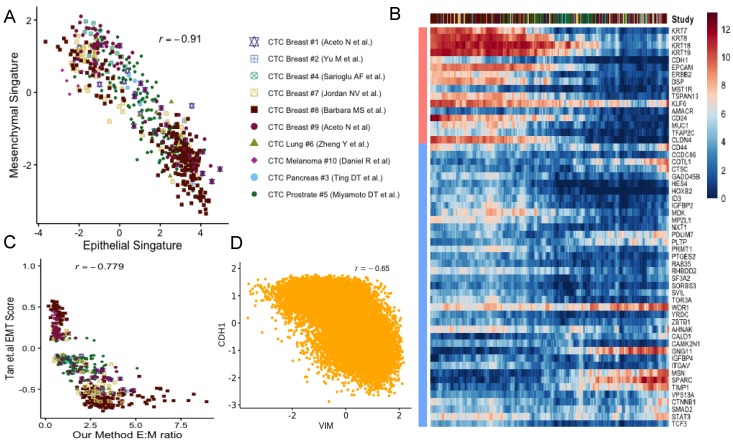
Epithelial-mesenchymal transition in cancer metastasis: (**A**) Scatter plot showing anti-correlation between epithelial and mesenchymal phenotypes across studies. (**B**) The moving average smoothen log(expression+1) of CTC dataset on epithelial and mesenchymal markers where cells are ordered based on their repctive E:M score as described in the main methods. (**C**) Scatter diagram depicting the correspondence between E:M score and the EMT score proposed by Tan and colleagues [40]. (**D**) CDH1-VIM anti-correlation observed due to simulation of EMT associated regulatory network.

Epithelial-mesenchymal transition (EMT) and mesenchymal-epithelial transition (MET) have long been postulated to play key roles in cancer metastasis and drug resistance [38]. The integration of CTC datasets presented us with the opportunity to probe into its validity. For each CTC, we computed two scores indicating the strength of epithelial and mesenchymal phenotypes respectively (Methods). In this analysis, we used tens of canonical markers of each of the concerned phenotypes. We detected near-perfect anti-correlation of (ρ = −0.91) the phenotypes across CTCs, coming from all cancer types (Figure 2A, Supplementary Figure S3). Our findings were consistent when we tracked the association between these phenotypes for CTCs from individual studies (Supplementary Figure S4). Notably, CTC transcriptomes were frequently found on a continuum of epithelial-mesenchymal transition in most of the datasets (Figure 2B). However, a agglomerative hierarchical clustering stratified the CTCs into two groups largely based on their approximate binarized identity as epithelial/mesenchymal cells (Supplementary Figure S13). In selected studies, in spite of being on a continuum, CTCs were found to form clusters towards the epithelial and the mesenchymal poles respectively (Supplementary Figure S4). Melanocytes derive from a highly invasive, multipotent embryonic cell population called the neural crest. It is suggested that the high degree of plasticity and the aggressiveness of malignant melanoma originate due to the re-activation of the embryonic neural crest program, which is silenced in due course of normal melanocyte differentiation [39].

Unlike the CTCs of most cancer types, circulating melanoma cells were found to be clustered exclusively around the mesenchymal pole of the E-M continuum (Supplementary Figure S4). Our E:M scores were found to be correlated (negatively) (ρ = −0.779) with EMT score as proposed by Tan and colleagues [40] (Figure 2C). One should note that a CTC, enriched with epithelial markers would receive a large positive E:M score, and a large negative EMT score. As a secondary validation, we constructed a network incorporating regulations among E and M genes under study (Methods, Supplementary Figure S5). Simulation experiments on this network using Ordinary Differential Equations (ODE) resulted in expression anti-correlation (ρ = −0.65) between *CDH1* and *VIM* (Methods, Figure 2D, Supplementary Figure S6).

### 3.3. Clear Patterns Observed in Expression Gradient of Immune Check-Point Inhibitor and Stemness Marker

**Figure 3:**
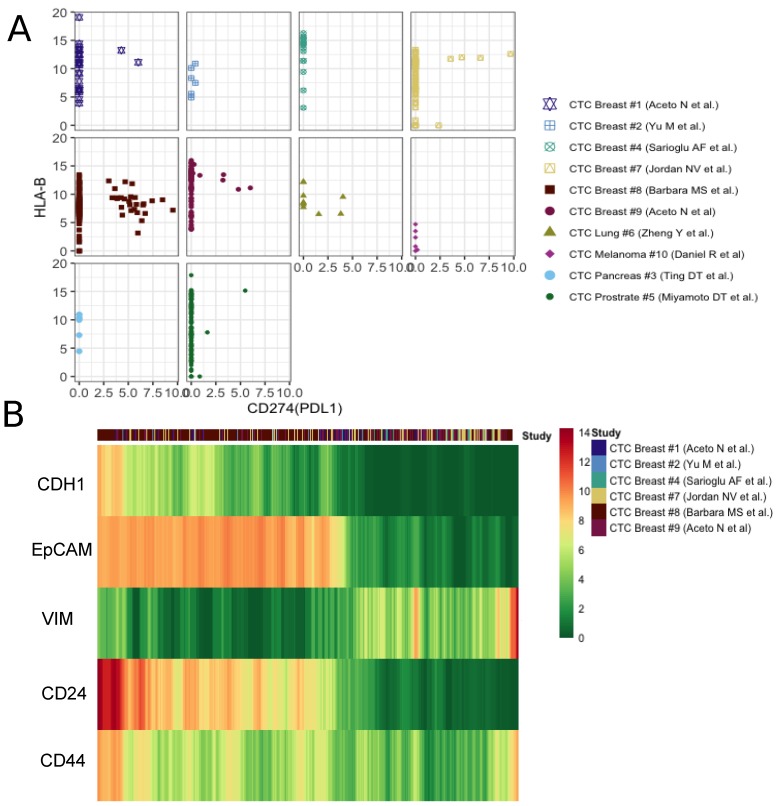
Patterns observed in expression gradient of immune check-point inhibitor and stemness markers. (**A**) The scatter plot of PDL1 and HLA-B expression in each study. (**B**) The moving average smoothen log(expression+1) of well known specific epithelial (CDH1,EpCAM), mesenchymal(VIM) and cancer stem cell markers (CD24, CD44) across breast CTCs, ordered based on the ratio of epithelial and mesenchymal signatures calculated as described in the main methods.

The activation of HLA class I (HLA-I) antigens on tumor cells is essential for the activation of cytotoxic T-lymphocytes. It has been demonstrated in mouse lines as well as human cancers that during natural cancer progression tumors gradually lose MHC-I expression as a result of a T-cell mediated immune selection [41]. On the other hand, the PD-1/PD-L1 pathway represents an adaptive immune resistance mechanism exerted by tumor cells in response to endogenous immune anti-tumor activity. PD-L1 expressed by tumor cells binds to PD-1 receptors on the activated T cells, which leads to the inhibition of the cytotoxic T cells [42]. Taken together, the loss of major histocompatibility complex (MHC) proteins (aka HLAs) and the activation of PD-L1 signify the prevention of cytotoxic T cell activities on tumor cells. Of late, immune checkpoint inhibitors, targeting the PD-1/PD-L1 pathway, have emerged as successful cancer treatment options [43]. In our curated datasets, we found only a minor fraction of CTCs expressing PD-L1. However, PD-L1-MHC anti correlation was evident across studies (Figure 3A). One of the datasets containing the maximum number of PD-L1-activated breast CTCs showed concurrence of PD-L1 with mesenchymal phenotype (Supplementary Figure S7). To date, multiple studies have linked EMT to the formation of cancer stem cells (CSCs). In a seminal paper, Mani and colleagues demonstrated the generation of a CD44^high^/CD24^low^, mammary stem cell-like population due to the induction of EMT. These cells were able to initiate tumors quite efficiently in the mouse. We tracked expression changes in CSC markers along E-M continuum [44]. CD44^high^/CD24^low^ CTCs indeed emerge late in the spectrum, following EMT induction (Figure 3b). This demonstrates how integrative analysis of CTC transcriptomes can help pinpoint stem-like phenotypes, with high tumorogenesis potential.

### 3.4. CTC-PBMC Classification System

**Figure 4:**
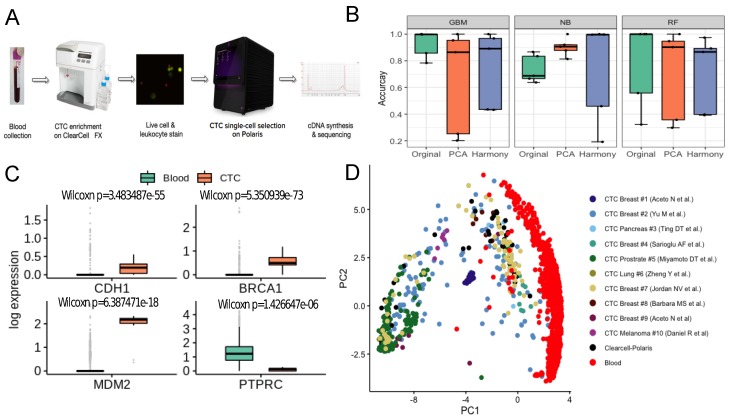
Label-free detection and characterisation of CTCs. (**A**) ClearCell-Polaris workflow involving size-based CTC enrichment by ClearCell FX system, followed by single cell selection and CD45/CD31 depletion using Polaris. (**B**) Performance of various machine learning algorithms in distinguishing between CTCs and PBMCs. Cells in each dataset were tested against a classifier trained on the remaining datasets. Box plots show the prediction accuracy’s for different choices of classification algorithms (Naive Bayes or NB, Random Forest or RF, Gradient Boosting Machine or GBM) and normalisation/batch-effect correction methods. (**C**) Box-plots showing canonical epithelial/breast cancer specific markers, up-regulated in the CTC population compared to the PBMCs. As expected, PTPRC, a pan leukocyte maker shows elevated expression levels in PBMCs as compared to CTCs. (**D**) Reference Component Analysis (RCA) based 2D projection of CTCs. PBMCs (red) are visibly separated from CTCs. CTCs enriched using the ClearCell-Polaris workflow cluster with CTCs of other types.

We trained a classifier on publicly available single cell expression profiles of human CTCs and PBMCs. Expression datasets curated from independent studies were subjected to rigorous data preprocessing steps (Methods). Notably, the state of the art batch effect removal method harmony [32] failed to improve the performance of the classification algorithms, compared to a simple median normalisation baseline (Supplementary Figure S12). We compared the performance of three classifiers—Naïve Bayes [33], Random Forest [35], and Gradient Boosting Machine [34]. We evaluated the model on five different datasets (**Methods**). Overall, the best performing model was GBM with a mean accuracy of ∼93% (Figure 4B). Notably, expression profiles of the CTCs retrieved by the Clearcell-Polaris system were all predicted as CTCs. ∼80% CTCs captured by the recently developed Hydro-Seq [11] (a hydrodynamic RNA-seq barcoding technique, for high-throughput CTC analysis) technique were classified as CTCs (Supplementary Table S4).

### 3.5. Identification of CTCs Captured Using Novel Label-Free Microfluidic Workflow

Existing technologies enrich CTCs with some level of contaminating white blood cells (WBCs). This poses a significant challenge in differentiating CTCs from immune cells. We addressed this challenge by integrating two commercially available microfluidic systems namely Biolidics ClearCell FX System [36] and the Fluidigm Polaris^TM^ system [19] (Methods, Figure 4A). In the proposed workflow CTCs are enriched in two steps - size-based enrichment by ClearCell, followed by CD45 (leukocyte marker) and CD31 (endothelial cell marker) based negative selection by Polaris [19].

To validate the workflow and the accompanying PBMC-CTC classification system, we processed peripheral blood samples of three HER2-, stage IV breast cancer patients (identified as P3, P4, P5) through the microfluidic device ensemble (Methods, Supplementary Figure S8). Polaris could retrieve 13, 12 and 32 cells from the blood samples of patients P3, P4, P5 respectively. 15 of these 57 cells passed the filtering criteria (Supplementary Figure S9). All 15 cells were classified as CTCs. We used additional validation criteria to determine the carcinogenic origin of the captured cells. When compared to a set of randomly selected PBMCs, ClearCell Polaris captured cells showed elevated expression of breast cancer-specific markers *BRCA1* and *MDM2* (*p*-value < 0.05) [45] (Figure 4C). We also detected up-regulation of *CDH1*, a canonical epithelial cell marker. Expression of *CD45* (PTPRC) was considerably low in these cells compared to the PBMC transcriptomes (*p*-value < 0.05) (Figure 4C). Reference component analysis (RCA) allows noise-free single cell clustering, by projecting single cell transcriptomes on reference bulk expression data. We subjected all CTC and PBMC transcriptomes to RCA analysis [37]. ClearCell-Polaris captured CTCs grouped with other CTCs, whereas the PBMCs formed a separate cluster (Methods, Figure 4d, Supplementary Figure S10).

## 4. Discussion

CTCs have been shown to be of prognostic significance in patients with various cancers [2,18,28]. We integrated single-cell expression profiles from various published studies and analyzed the emergence of epithelial to mesenchymal transition among CTCs. For this, we developed the E:M score that ordered CTC transcriptomes on an approximate pseudo-temporal axis of epithelial-mesenchymal transition. Our proposed EMT scoring method, in principle, is similar to the method proposed by Tan and colleagues, which focuses on six major cancer types, namely ovarian, breast, bladder, colorectal, gastric, and lung. Different from this, we used widely accepted, literature curated E and M markers agnostic of the cancer types. Although both the methods correlate well when applied to the CTC transcriptomes (Figure 2C), we found our proposed methods depict the E to M continuum better (Figure 2B and Supplementary Figure S14).

It is suspected that a large number of CTCs do not portray the signature of cancer epithelium, largely due to their acquired phenotype that is suitable for migration [28]. We leveraged the power of machine learning in techniques in reliably distinguishing CTCs from other relatively way more abundant immune cell types. This is achieved by the integration of publicly available CTC datasets and machine learning-based model training. We provide a user-friendly R package for CTC classification that provides a probabilistic score indicating the cancer origin of individual cells. Our reported ClearCell^®^ Polaris^™^ workflow, in tandem with the machine learning based CTC-immune cell classification system, for the first time, enables truly unbiased detection of circulating tumor cells. With declining per cell cost associated with single-cell gene expression screening, we speculate a high adoption rate for our proposed strategy.

An integrative study of CTC transcriptomes presented us with the opportunity to discover consistent pan-cancer CTC surface-proteins, besides *EpCAM*. We looked for surface-protein coding genes that are deferentially upregulated in CTCs over blood cells (Supplementary Note-3). Most remarkable among these were *ITGB5, TACSTD2, SLC39A6* (Supplementary Figure S12). In addition to *EpCAM*, some of these markers might be useful to broad-base marker dependent capture of CTCs.

## Supplementary Materials

The following are available online at https://www.mdpi.com/2077-0383/9/4/1206/s1, Supplementary Note 1: Network analysis to investigate the mechanistic basis of EMT continuum phenotype observed in the data analysis, Supplementary Note 2: Gene expression quantification of CTCs detected by the ClearCell Polaris workflow, Supplementary Note 3: Exploration of novel surface markers for CTCs, Supplementary Figure S1: Data Quality of studies, Supplementary Figure S2: Expression of known markers in curated CTCs and PBMCs, Supplementary Figure S3: Combined epithelial, mesenchymal and cancer stem cell signatures, Supplementary Figure S4: Scatter plots show Epithelial-Mesenchymal anti-correlation for individual datasets, Supplementary Figure S5: The network simulated using RACIPE, Supplementary Figure S6: Random network simulation results, Supplementary Figure S7: Expression Gradient of Immune Check-Point Inhibitor and Stemness Marker, Supplementary Figure S8: Treatment history of the patients, Supplementary Figure S9: Number of expressed genes in CTCs detected using the Clearcell-Polaris workflow. Supplementary Figure S10: Tissue - single cell correlation plot obtained from RCA, Supplementary Figure S11: Log2 fold change of surface markers between CTC and PBMC populations, Supplementary Figure S12: PCA plots of log transformed median normalized counts and Harmony batch correction method, Supplementary Figure S13: Clustered heatmap of Main Figure 2B, Supplementary Figure S14: Continuum plot using Tan et al method, Supplementary Table S1: List of all studies from which datasets are used, Supplementary Table S2: Functional details of the EMT related genes used in the study, Supplementary Table S3: Genes used as features for machine learning based analyses, Supplementary Table S4: Machine learning results.
