# Erratum: Iyer, A., et al. Integrative Analysis and Machine Learning Based Characterization of Single Circulating Tumor Cells. *J. Clin. Med.* 2020, *9*, 1206

**DOI:** 10.3390/jcm10020370

**Published:** 2021-01-19

**Authors:** Arvind Iyer, Krishan Gupta, Shreya Sharma, Kishore Hari, Yi Fang Lee, Neevan Ramalingam, Yoon Sim Yap, Jay West, Ali Asgar Bhagat, Balaram Vishnu Subramani, Burhanuddin Sabuwala, Tuan Zea Tan, Jean Paul Thiery, Mohit Kumar Jolly, Naveen Ramalingam, Debarka Sengupta

**Affiliations:** 1 Department of Computational Biology, Indraprastha Institute of Information Technology, New Delhi 110020, India; arvind16122@iiitd.ac.in; 2 Department of Computer Science and Engineering Indraprastha Institute of Information Technology, New Delhi 110020, India; krishang@iiitd.ac.in (K.G.); shreya15096@iiitd.ac.in (S.S.); 3 Centre for BioSystems Science and Engineering, Indian Institute of Science, Bangalore 560012, India; kishorehari@iisc.ac.in (K.H.); mkjolly@iisc.ac.in (M.K.J.); 4 Biolidics Limited, 81 Science Park Drive, 02-03 The Chadwick, Singapore 118257, Singapore; yifang@biolidics.com (Y.F.L.); ali@nus.edu.sg (A.A.B.); 5 Qualcomm Incorporated, 5775 Morehouse Drive, San Diego, CA 92121, USA; neevan.snap@gmail.com; 6 National Cancer Centre, 11 Hospital Dr, Singapore 169610, Singapore; Yap.Y.S@nccs.com.sg; 7 Fluidigm Corporation, 2 Tower Place, Suite 2000, South San Francisco, CA 94080, USA; drjayman@gmail.com; 8 School of Mathematics, Indian Institute of Science Education and Research, Thiruvananthapuram 695551, India; balaram.vishnu17@iisertvm.ac.in; 9 Department of Biotechnology, Indian Institute of Technology Madras, Chennai 600036, India; be17b011@smail.iitm.ac.in; 10 Cancer Science Institute of Singapore, National University of Singapore, Center for Translational Medicine, Singapore 117599, Singapore; csittz@nus.edu.sg; 11 Guangzhou Regenerative Medicine and Health; Guangdong laboratory, Guangzhou 510530, China; bchtjp@nus.edu.sg; 12 Center for Artificial Intelligence, Indraprastha Institute of Information Technology, New Delhi 110020, India

In the published manuscript [1], the bibliographic items were incorrectly rendered. The following three references are missed [2,3,4]. Due to this, the order and the citations corresponding to several references were misplaced. We added them between Szczerba, B.M., et al. (2019) and Zhang, X. (2018) and numbered them as [29–31] in the original manuscript [1]. The citation has now been inserted in ***Section 2***. ***Materials and Methods***, ***2.2. Data Pre-Processing***, ***Paragraph 1***. The subsequent citations in the main text have also been updated. 

The authors apologize for any inconvenience caused and state that the scientific conclusions are unaffected. The original article has been updated.
