# Supplementary material for: Integrative Analysis and Machine Learning based Characterization of Single Circulating Tumor Cells
Source: J Clin Med. 2020 Apr 22;9(4):1206. doi: 10.3390/jcm9041206 (PMC7230872; doi:10.3390/jcm9041206)
Supplement: Supplementary file 1 [file jcm-09-01206-s001.zip › supplementary_proofread.pdf]

Supplementary Data

Integrative analysis and machine learning based characterization of single circulating tumor cells

Arvind Iyer[1,†,¶], Krishan Gupta[2,†], Shreya Sharma[2], Kishore Hari[3], Yi Fang Lee[4], Neevan Ramalingam[5], Yoon Sim Yap[6], Jay West[7,‡], Ali Asgar Bhagat[4,§,‖], Balaram Vishnu Subramani[8], Burhanuddin Sabuwala[9], Tuan Zea Tan[10], Jean Paul Thiery[11], Mohit Kumar Jolly[3], Naveen Ramalingam[7,*], and Debarka Sengupta[1,2,12,*]

1] Department of Computational Biology, Indraprastha Institute of Information Technology, New Delhi, 110020, India
2] Department of Computer Science and Engineering Indraprastha Institute of Information Technology, New Delhi, 110020, India.
3] Centre for BioSystems Science and Engineering, Indian Institute of Science, Bangalore, 560012, India.
4] Biolidics Limited, 81 Science Park Drive, 02-03 The Chadwick, Singapore 118257, Singapore.
5] Qualcomm Incorporated, 5775 Morehouse Drive, San Diego, CA 92121, USA.
6] National Cancer Centre, 11 Hospital Dr, Singapore 169610, Singapore.
7] Fluidigm Corporation,2 Tower Place, Suite 2000,,South San Francisco, CA 94080, USA
8] School of Mathematics, Indian Institute of Science Education and Research, Thiruvananthapuram, 695551, India.
9] Department of Biotechnology, Indian Institute of Technology Madras, Chennai 600036, India.
10] Cancer Science Institute of Singapore, National University of Singapore, Center for Translational Medicine, 117599, Singapore.
11] Guangzhou Regenerative Medicine and Health; Guangdong laboratory, Guangzhou 510530, China.
12] Center for Artificial Intelligence, Indraprastha Institute of Information Technology, New Delhi, 110020, India.

‡ Current address: BioSkryb Corporation, BioLabs, 701 W Main St, Suite 200, Durham, NC 27701, USA.
§ Current address: Department of Biomedical Engineering, Faculty of Engineering, National University of Singapore, Engineering Drive 1, Singapore 117575, Singapore.
‖ Current address: Institute for Health Innovation and Technology (iHealthtech), National University of Singapore, 14 Medical Drive, Singapore 117599, Singapore
¶ Current address: Department of Computational Biology, University of Lausanne (UNIL), Lausanne 1015, Switzerland.
†First Author
* To whom correspondence should be addressed.Tel: +9111 26907446; Emails: naveen.ramalingam@fluidigm.com, debarka@iiitd.ac.in

Supplementary Note 1: Network analysis to investigate the mechanistic basis of EMT continuum phenotype observed in the data analysis.

To investigate the mechanistic basis of our data analysis from multiple CTC datasets, we explored the expansive literature for a functional implication of the genes that were identified in epithelial and mesenchymal signatures for their roles in EMT and/or MET (see Supplementary Table 2). We next constructed a network based on the functional implications of these genes in EMT and/or MET, including their effects on the regulatory feedback loops involving miR-200, ZEB and GRHL2 – the fulcrum of epithelial-mesenchymal plasticity. We mapped the connections known to promote EMT and metastasis (SNAIL, TIMP1, etc.) through promoting ZEB and/or inhibiting miR-200. Moreover, some genes in this list have already been known to have a direct effect on CDH1 and VIM. Please note that many of the molecules identified here are markers of epithelial and mesenchymal state, and their functional impact on EMT or metastasis remains elusive, hence they were excluded from the network. CDH1 and VIM were chosen to denote epithelial and mesenchymal states respectively; their expression levels are considered as the outcome for the network.

As the data were collected across multiple cancer types, the parameters for each connection are very likely to vary. Hence, instead of applying a single parameter set to the network, we sampled the parameter space via a uniform distribution using a tool called RAndom CIrcuit PErturbation (RACIPE)[1]. As the name suggests, RACIPE[1] chooses the parameter space of a given circuit randomly to elucidate the robust dynamical outcomes of the network and pinpoint the gene expression signatures most likely to emerge from the given network topology.

To understand the significance of the network topology, we generated random network topologies by swapping the edges while maintaining the degree of each node and the number of activating and inhibiting edges in the network, ensuring the conservation of the nature of the nodes. Furthermore, we characterized the effect of single edge perturbations (SEP's), i.e., change in the sign of one edge in the network at a time, on the correlation between ZEB-miR200 and VIM-CDH1 pairs. With randomized networks (Supplementary Fig-ta,Supplementary Fig-6a), we observe that the correlation between the markers VIM-CDH1 as well as the core elements ZEB-miR200 is strongly negative in the original network, denoted by wildtype (WT). A very small fraction of randomly generated topologies have equal or stronger correlation than WT for both (CDH1, VIM) and (ZEB, miR-200), suggesting the importance of the particular network topology for the observed behavior. Similarly, we observe that most SEPs do not show as a strong correlation in terms of (CDH1, VIM) or (ZEB, miR-200) as the original network (WT, shown in red). The effect of perturbations on the network is calculated by applying a distance metric, Jensen-Shannon Divergence (JSD)[2], on the steady-state frequencies of the networks.

Supplementary Note 2: Gene expression quantification of CTCs detected by the ClearCell Polaris workflow

An index for RNA-Seq by expectation maximization (RSEM) was generated based on the hg19 RefSeq transcriptome downloaded from the UCSC Genome Browser database. Read data were aligned directly to this index using RSEM/bowtie. Quantification of gene expression levels in counts for all genes in all samples was performed using RSEM v1.2.4[3]. Genomic mappings were performed with TopHat 2 v2.0.13[4], and the resulting alignments were used to calculate genomic

mapping percentages. Raw sequencing read data were aligned directly to the human rRNA sequences NR_003287.1 (28s), NR_003286.1 (18S) and NR_003285.2 (5.8S) using bowtie 2 v2.2.4[5], and the percentage of reads aligned to rRNA was then calculated as reads aligned to these sequences divided by the total reads.

Supplementary Note 3: Exploration of novel surface markers for CTCs.

We performed Wilcoxon's rank-sum test for determining differentially expressed genes between CTCs and blood cells. P-values thus obtained were subjected to multiple test corrections using the Benjamin-Hochberg method (p.adjust function in R). We applied an FDR cut off of 0.05 for selecting the differential genes (DE). DE genes that were expressed in at least 80% of the CTCs were retained. We downloaded a list of the surface proteins from the Cell Surface Protein Atlas (CSPA) database[6] and took intersection with the narrowed set of DE genes. Supplementary Fig-12 displays the selected markers in the order of the gene-wise fold change values.

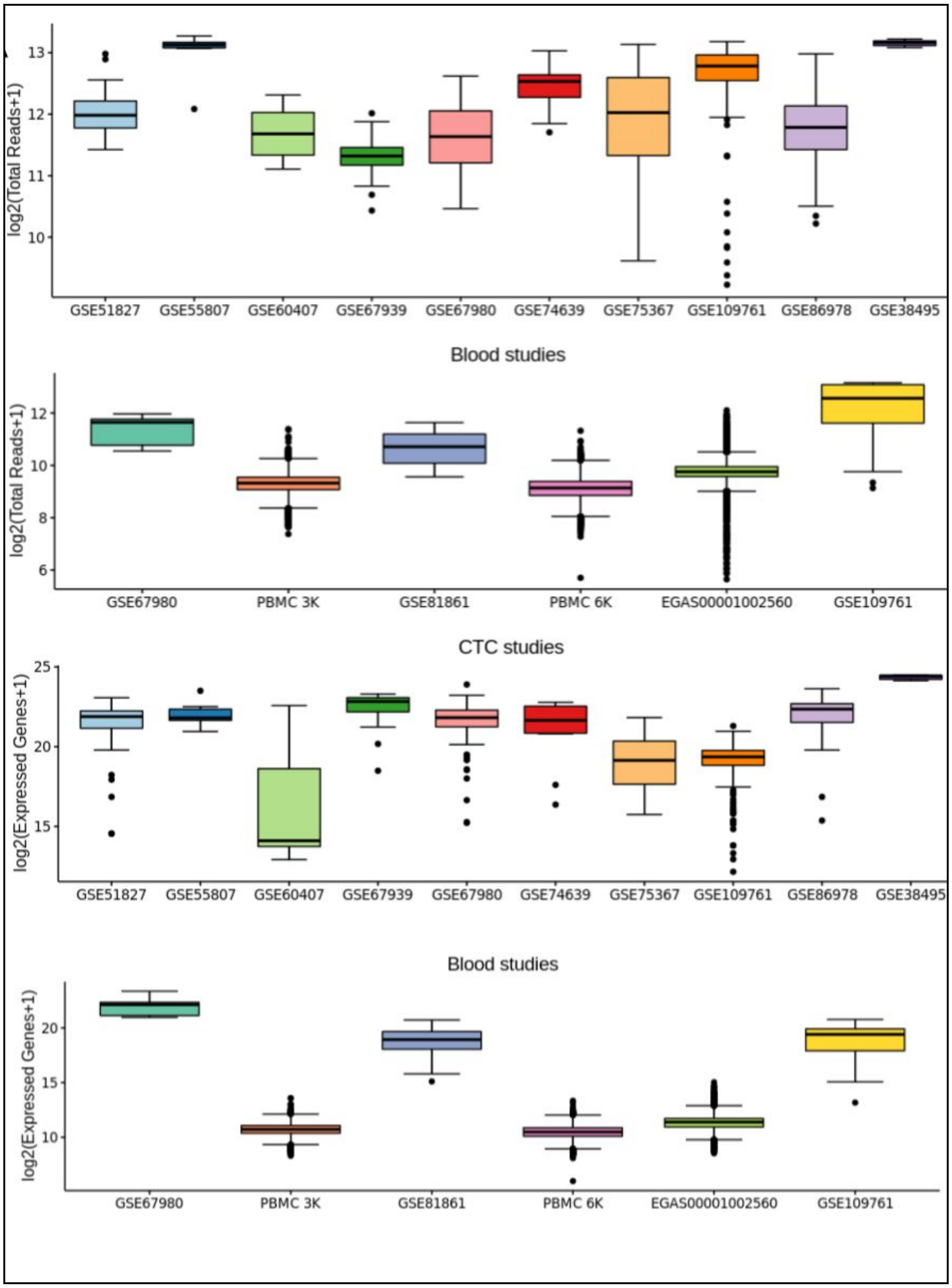

Supplementary Figure-1: A) Boxplots show the distribution of total read counts across cells in each dataset. B) Boxplots show the distribution of the number of detected (non zero) genes in each dataset.

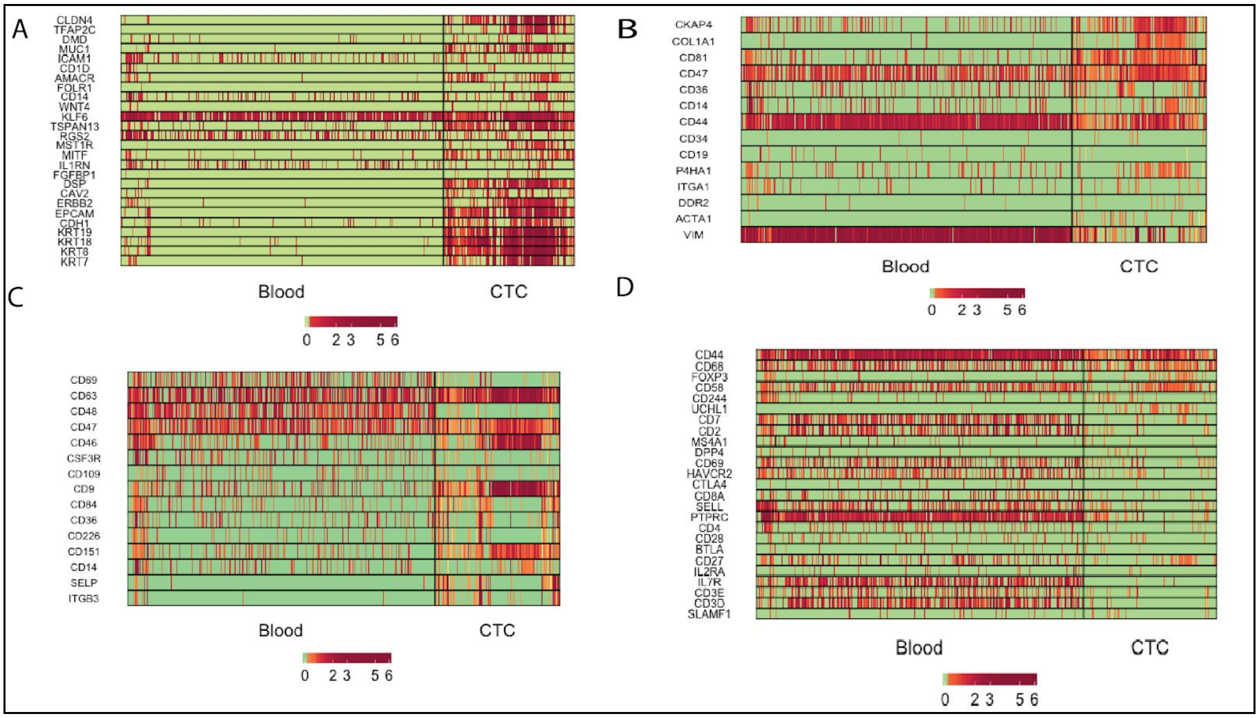

Supplementary Figure-2: Expression of known markers in curated CTCs and PBMCs
A) Expression of Epithelial markers in the integrated dataset of CTCs and PBMCs (blood). B) Expression of Fibroblast markers C) Expression of Platelet markers. D) Expression of T-cell markers.

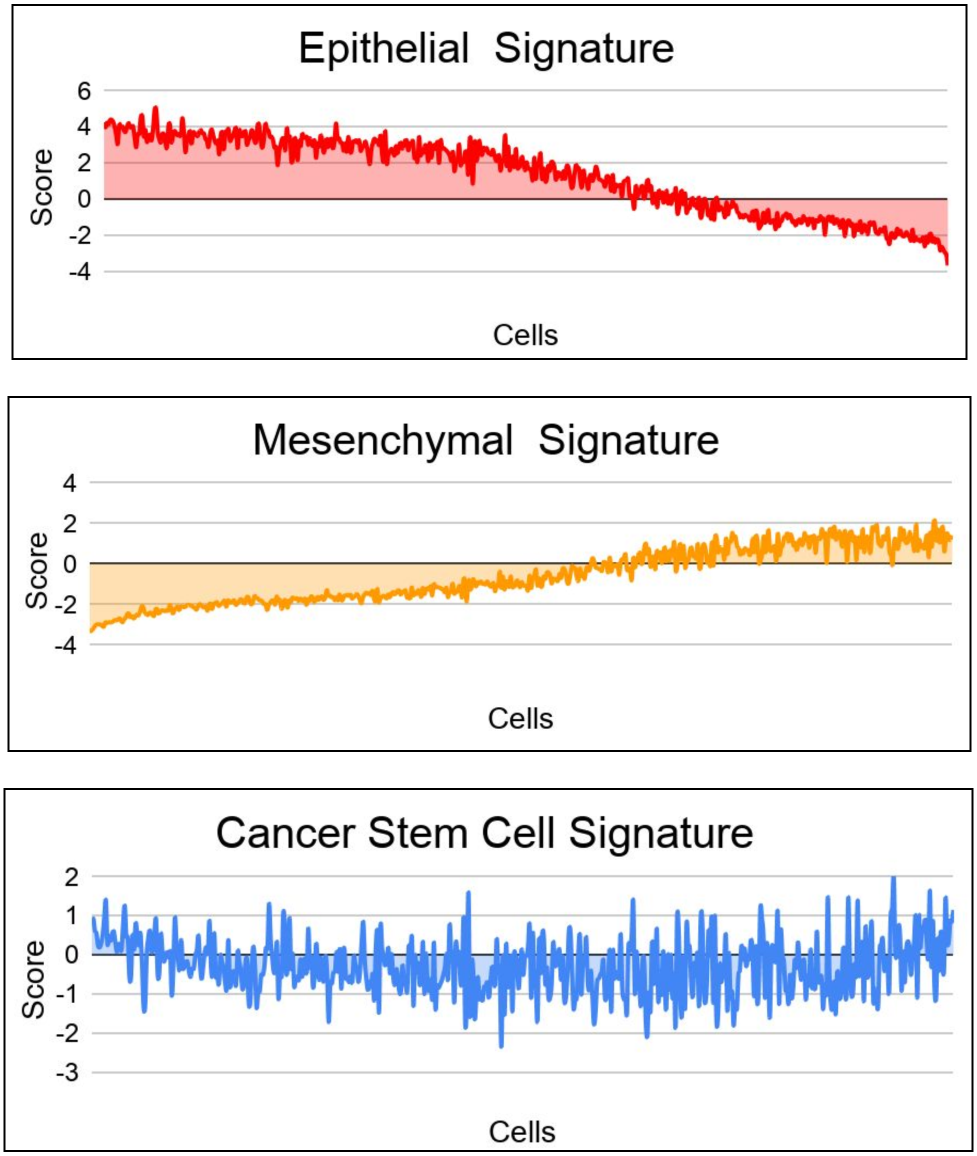

Supplementary Figure-3: Combined epithelial, mesenchymal and cancer stem cell signatures.

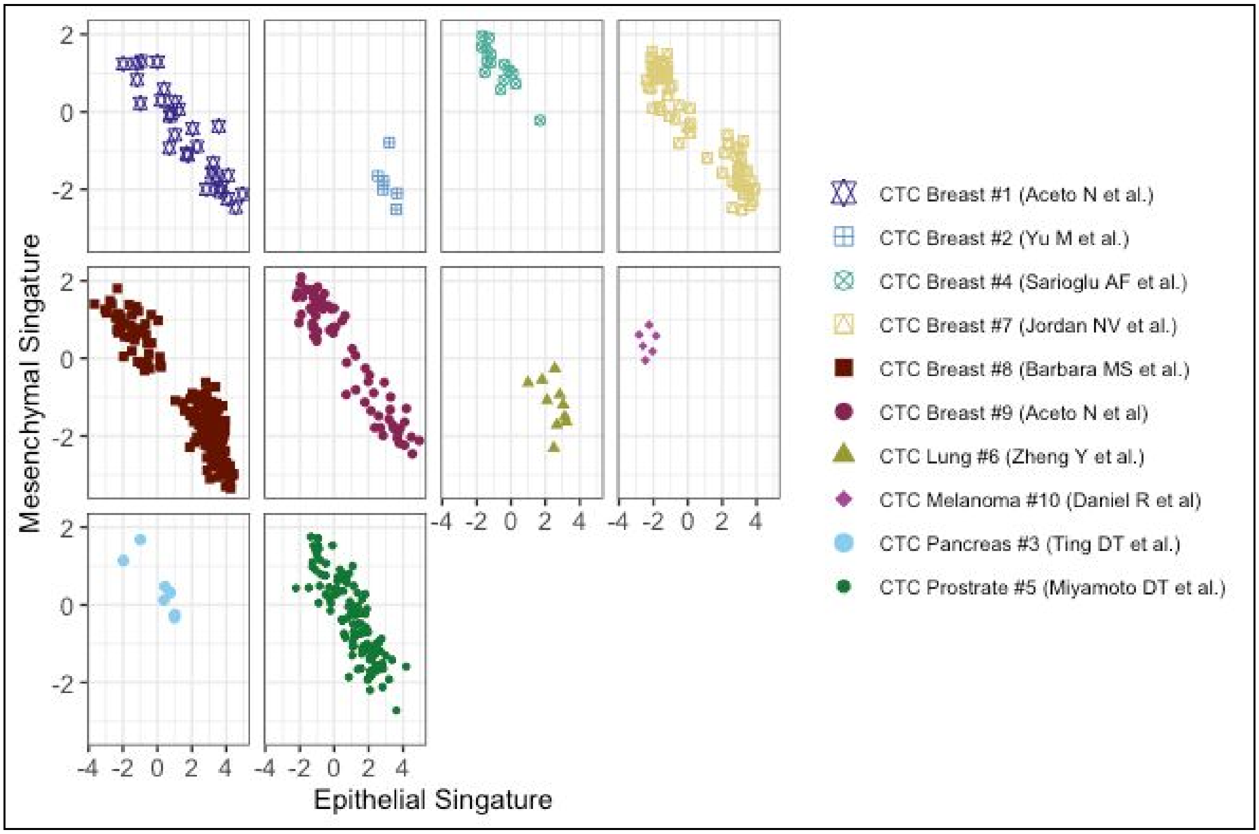

Supplementary Figure-4: Scatter plots show Epithelial-Mesenchymal anti-correlation for individual datasets.

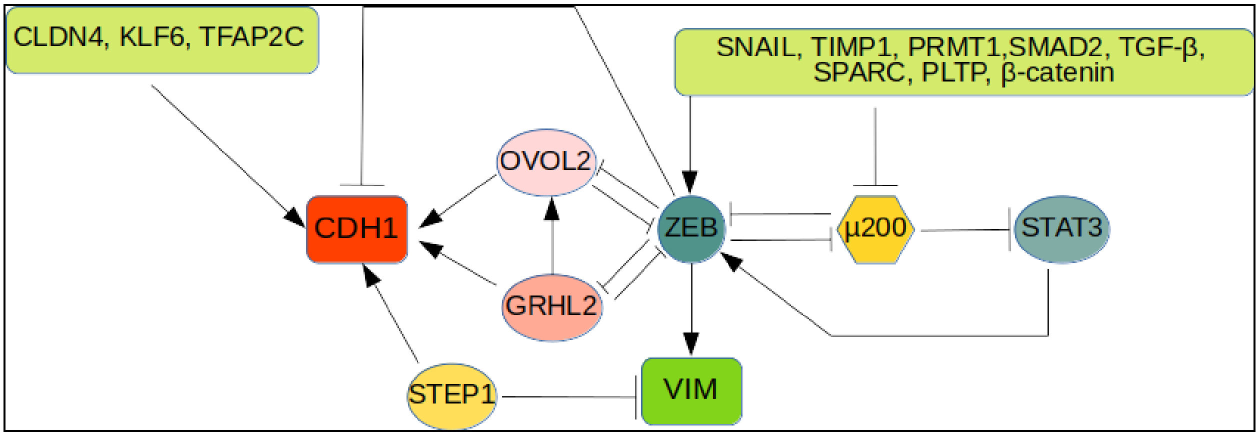

Supplementary Figure-5: The network simulated using RACIPE, including genes used for the creation of epithelial and mesenchymal signatures. Here µ200 represents miRNA-200.

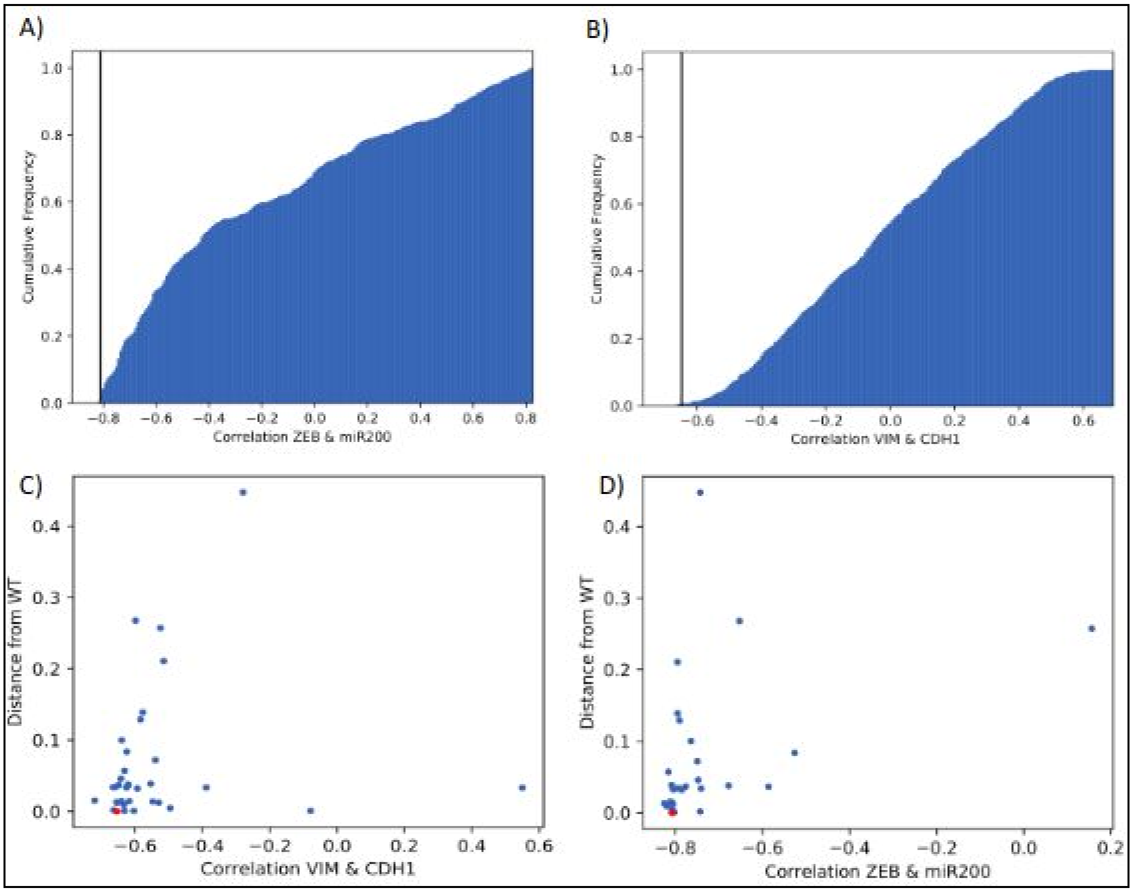

Supplementary Figure-6: Top: Cumulative frequency distribution of the correlation between A) VIM and CDH1 and B) ZEB and miR200. The black line represents the correlation coefficient for 'wildtype' (WT) network. Bottom: scatter plots of JSD distance against the correlation between C) VIM and CDH1 and D) ZEB and miR200 for SEP's. Y-axis represents the distance of each SEP from WT (JSD).

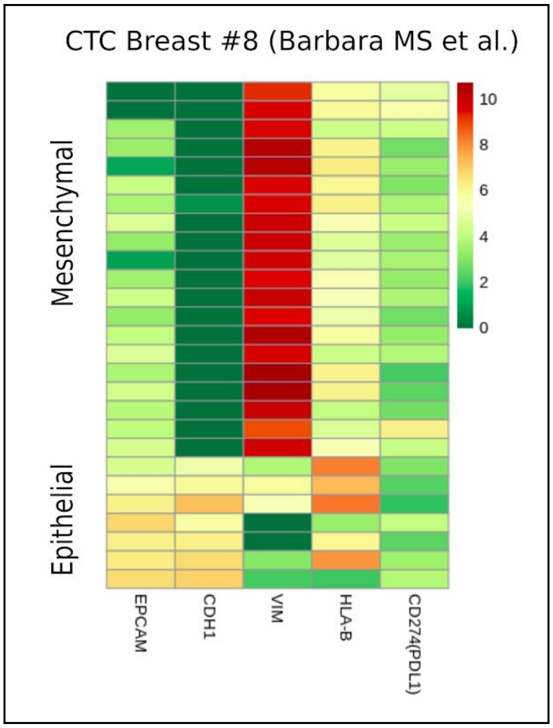

Supplementary Figure-7: The heatmap of $\log_e$ (expression+1) of selected epithelial, mesenchymal marker along with PDL1 and HLA-B for cells with non zero PDL1 expression from one specific study (having maximum numbers of PDL1 expressing cells). Note that we only showed the HLA-B expression since this was most well expressed.

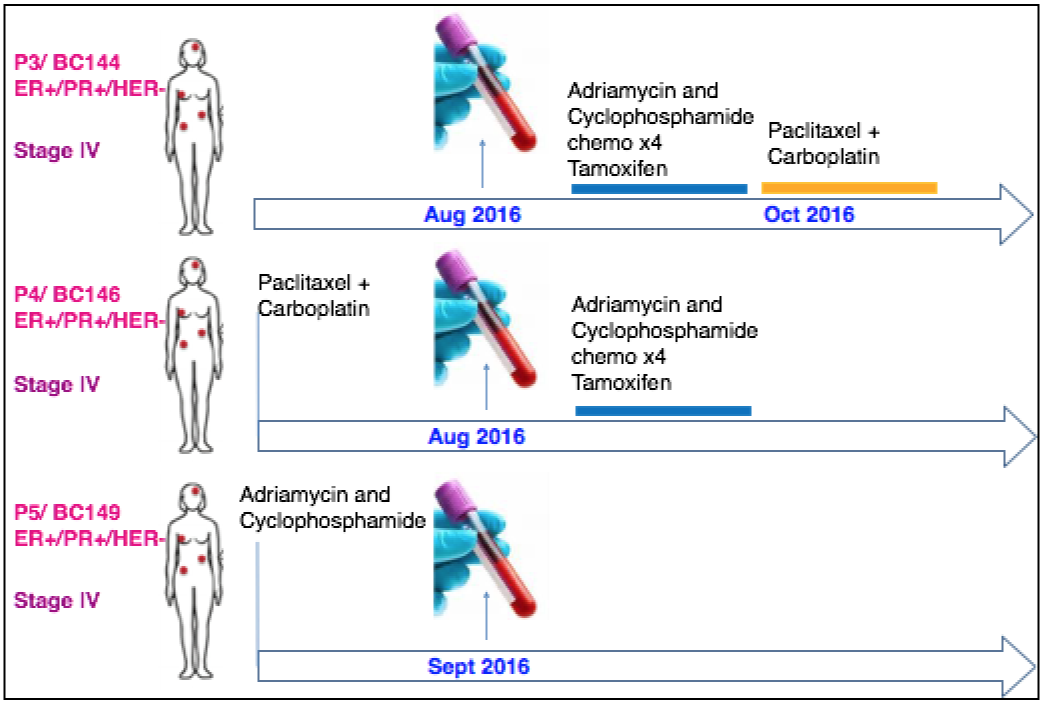

Supplementary Figure-8: Treatment history of the patients

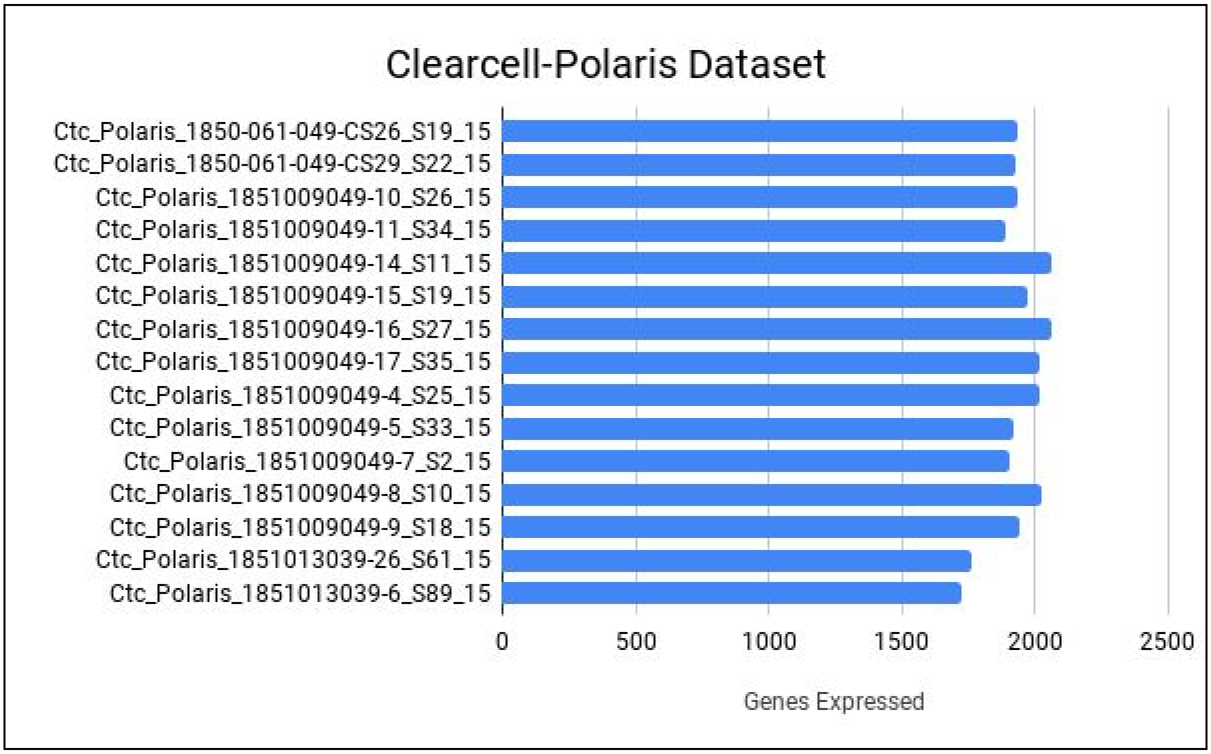

Supplementary Figure-9: Number of expressed genes in CTCs detected using the Clearcell-Polaris workflow

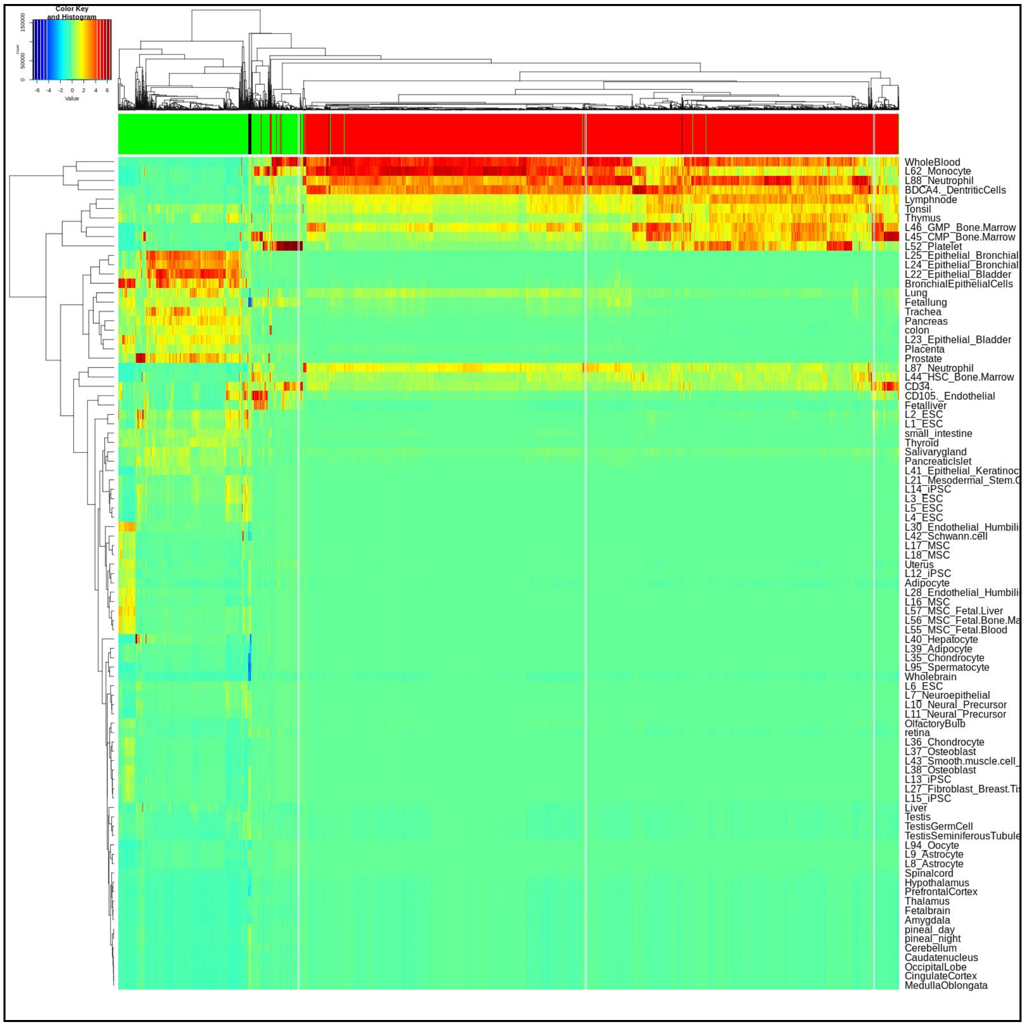

Supplementary Figure-10: Tissue - single cell correlation plot obtained from RCA

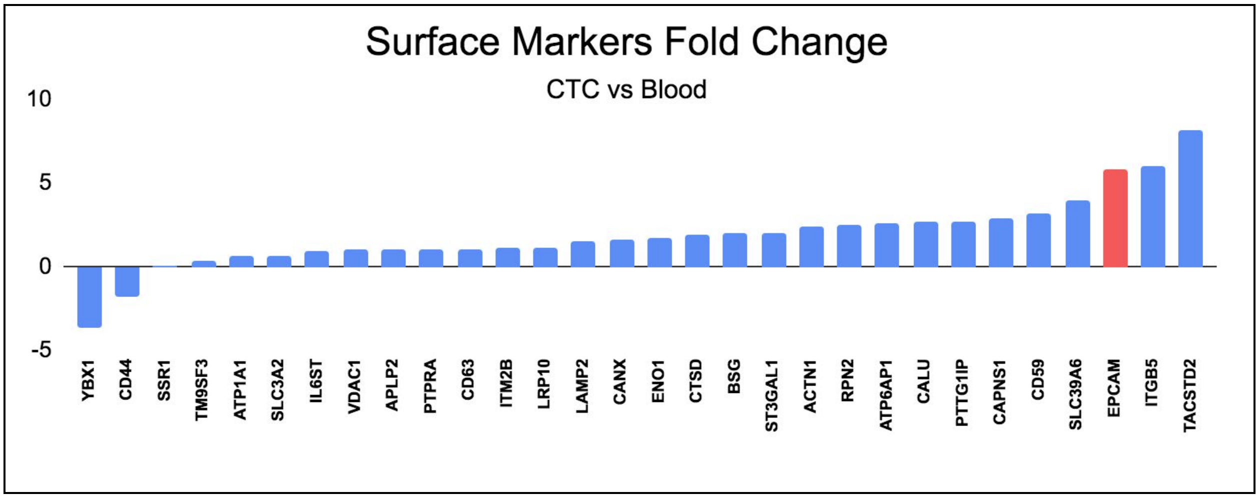

Supplementary Figure-11: Log2 fold change of surface markers between CTC and PBMC populations. Besides EpCAM, few genes including ITGB5, TACSTD2, SLC39A6 appear specific to CTCs.

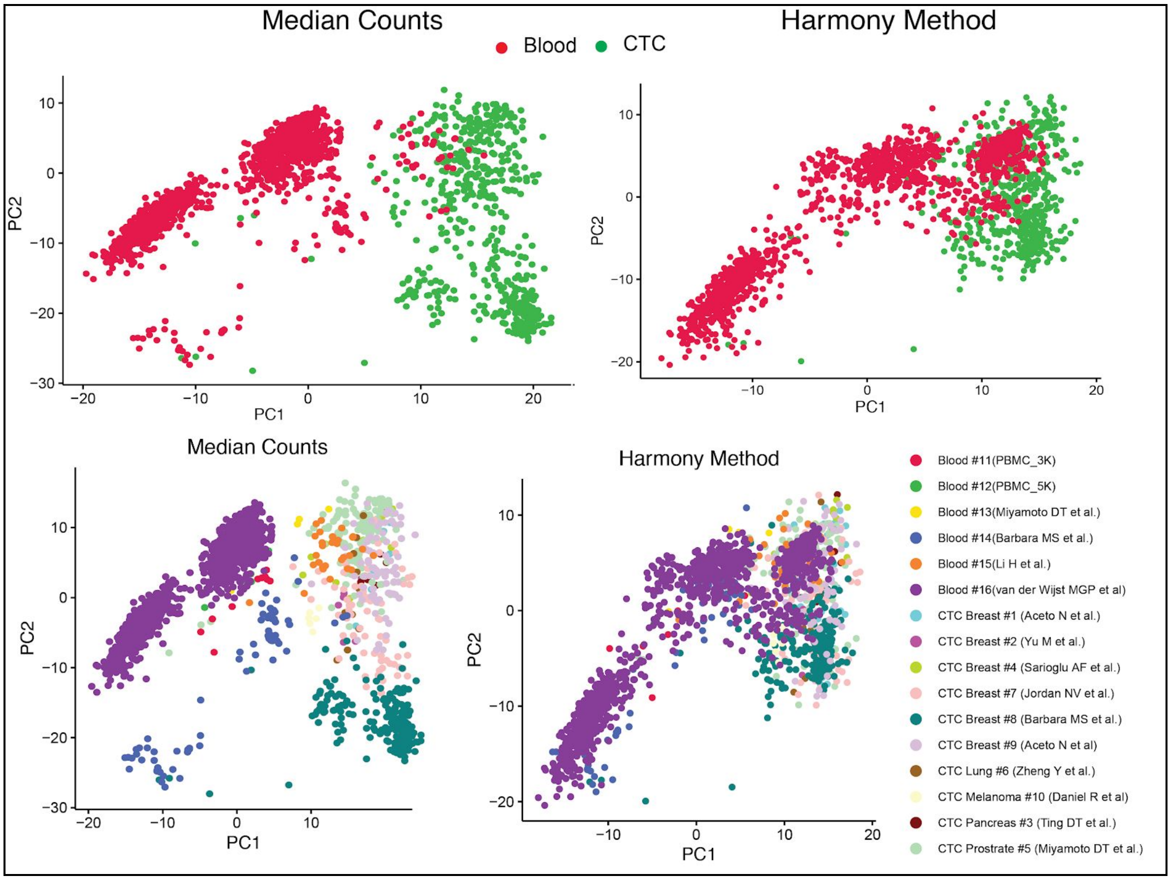

Supplementary Figure-12: PCA plots of log transformed median normalized counts and Harmony batch correction method.

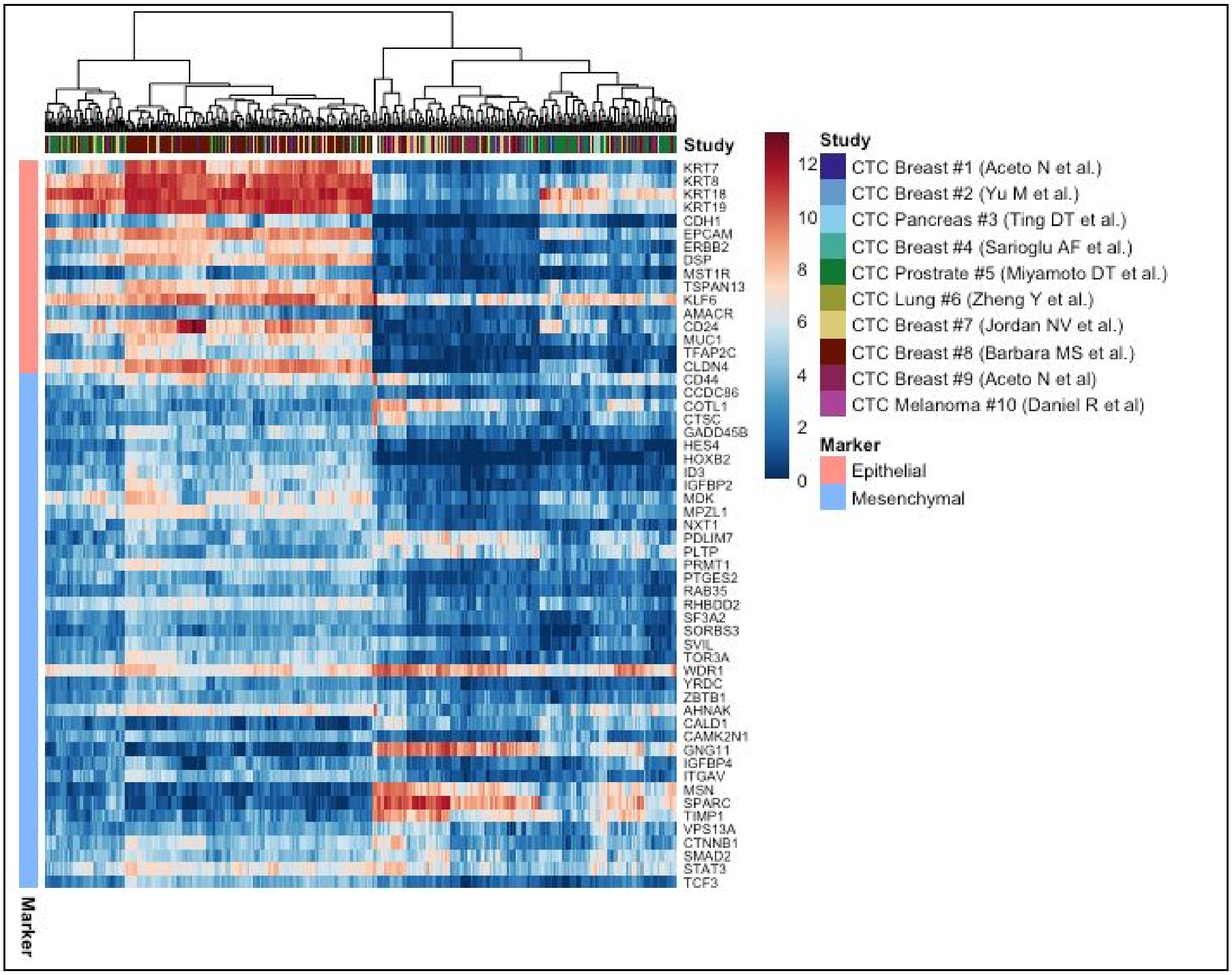

Supplementary Figure-13: Clustered heatmap of Main Figure 2-B

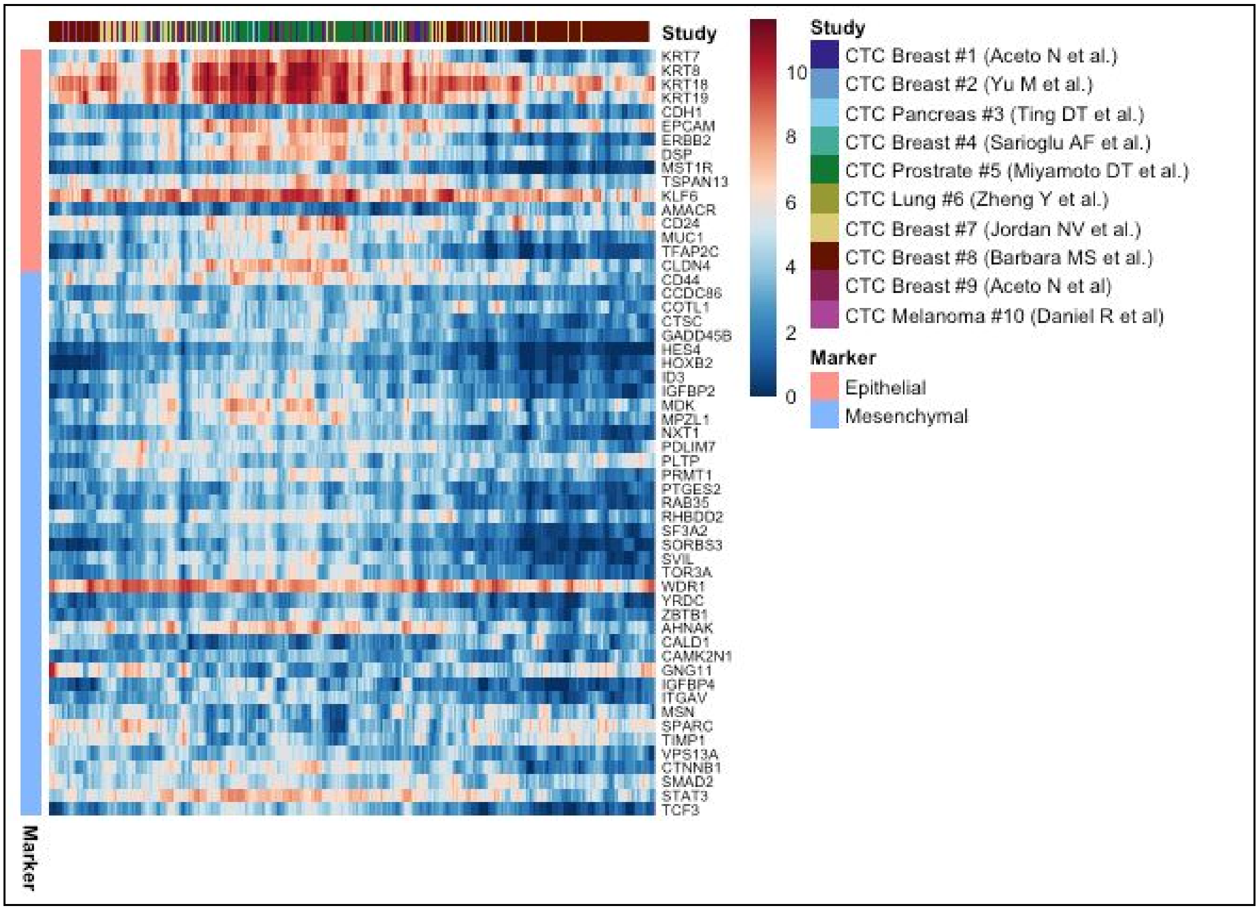

Supplementary Figure-14: Continuum plot using Tan et al method.

Supplementary Table 1. List of all studies from which datasets are used

The table can be found as a separate file **Supplementary_Table_1.xlsx.** The sheet contains data of the studies used in the project in the form of a table with identifier, title, number of samples, link etc. More information about the studies can be fetched from the links provided.

Supplementary Table 2. Functional details of the EMT related genes used in the study.

The table can be found as a separate file **Supplementary_Table_2.xlsx.** The sheet contains data of the genes used for all the analysis related to EMT.

Supplementary Table 3. Genes used as features for machine learning based analyses.

The table can be found as a separate file **Supplementary_Table_3.xlsx.** The sheet contains data of the list of genes used as features for machine learning model.

Supplementary Table 4. Machine learning results The table can be found as a separate file **Supplementary_Table_4.xlsx** The workbook **Supplementary_Table_4.xlsx** contains testing and training statistics done on three ml models.

References:

1] Huang, Bin, et al. "Interrogating the topological robustness of gene regulatory circuits by randomization." *PLoS computational biology* 13.3 (2017): e1005456.
2] Lin, Jianhua. "Divergence measures based on the Shannon entropy." *IEEE Transactions on Information theory* 37.1 (1991): 145-151.
3] Li, Bo, and Colin N. Dewey. "RSEM: accurate transcript quantification from RNA-Seq data with or without a reference genome." *BMC Bioinformatics* 12.1 (2011): 323.
4] Kim, Daehwan, et al. "TopHat2: accurate alignment of transcriptomes in the presence of insertions, deletions and gene fusions." *Genome biology* 14.4 (2013): R36.
5] Langmead, Ben, and Steven L. Salzberg. "Fast gapped-read alignment with Bowtie 2." *Nature methods* 9.4 (2012): 357.
6] Bausch-Fluck, Damaris, et al. "A mass spectrometric-derived cell surface protein atlas." *PloS one* 10.4 (2015): e0121314.
